# Polymeric nanoparticles for dopamine and levodopa replacement in Parkinson's disease

**DOI:** 10.1039/d2na00524g

**Published:** 2022-11-03

**Authors:** Obaydah Abd Alkader Alabrahim, Hassan Mohamed El-Said Azzazy

**Affiliations:** Graduate Nanotechnology Program, School of Sciences & Engineering, The American University in Cairo 11835 New Cairo Egypt obaydah.alabrahim@aucegypt.edu; Department of Chemistry, School of Sciences & Engineering, The American University in Cairo AUC Avenue, P. O. Box 74 New Cairo 11835 Egypt hazzazy@aucegypt.edu; Department of Nanobiophotonics, Leibniz Institute for Photonic Technology Albert Einstein Str. 9 Jena 07745 Germany hassan.azzazy@leibniz-ipht.de

## Abstract

As the world's population ages, the incidence of Parkinson's disease (PD), the second most common neurological ailment, keeps increasing. It is estimated that 1% of the global population over the age of 60 has the disease. The continuous loss of dopaminergic neurons and the concomitant brain depletion of dopamine levels represent the hallmarks of PD. As a result, current PD therapies primarily target dopamine or its precursor (levodopa). Therapeutic approaches that aim to provide an exogenous source of levodopa or dopamine are hindered by their poor bioavailability and the blood–brain barrier. Nevertheless, the fabrication of many polymeric nanoparticles has been exploited to deliver several drugs inside the brain. In addition to a brief introduction of PD and its current therapeutic approaches, this review covers novel polymeric nanoparticulate drug delivery systems exploited lately for dopamine and levodopa replacement in PD.

## Abstract 2

This review focuses on the recent polymeric nanoparticulate drug delivery systems that have been exploited for dopamine and levodopa replacement in Parkinson's disease.

## Graphical abstract

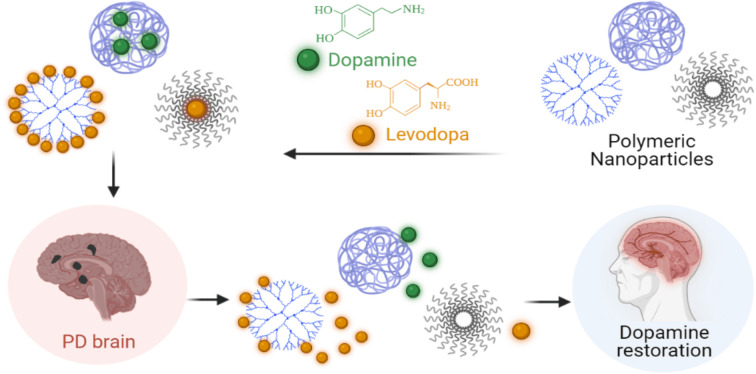

## 1. Introduction

**Fig. 1:**
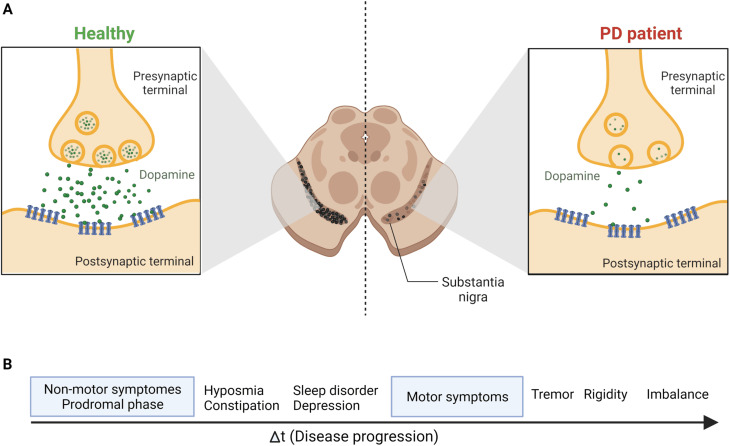
The continuous loss of dopaminergic neurons in SNpc leads to the prevalence of the most common PD symptoms. (A) The release of the DA neurotransmitter from the synaptic terminals of striatal neurons, as shown above, indicates the gradual and permanent loss of DA in PD patients because of the nigrostriatal degeneration. (B) Disease progression and symptoms development. The prodromal phase, the non-motor symptoms phase, may last for 20 years before the motor symptoms start to develop.^69^ Figure drawn using Biorender.

Parkinson's disease (PD) is a worldwide major public health concern defined as one of the most common neurodegenerative disorders, the second to Alzheimer's disease.^1,2^ Neurodegenerative disorders are classified as a group of neurological ailments that form specific brain lesions which develop over time. Such brain lesions combined with the gradual loss of the neurocentral regulation of the affected individuals are responsible for the deteriorating symptoms among patients.^3,4^ These symptoms are due to the severe loss of dopamine (DA), one of the key neurotransmitters, from the striatum, which affects the nigral pathway of the substantia nigra pars compacta (SNpc), causing dopaminergic neurons to degenerate irreversibly and progressively (by 40–50%). Also, 80% to 90% DA depletion was reported.^5^ Consequently, PD's main clinical manifestations include a triangle of motor symptoms, namely resting tremors, bradykinesia/akinesia, and muscular rigidity.^1,3^ Other non-motor symptoms include depression, anxiety, gastrointestinal disorders, olfactory and visual abnormalities, weight loss, cognitive deficits, *etc.*^6–8^ (Fig. 1).

Due to their physicochemical nature, most of the pharmacological therapies that are currently prescribed to manage the main symptoms of PD have poor bioavailability (BAV) and pharmacokinetic profiles and are incapable of diffusing across the blood–brain barrier (BBB). In addition, diagnosed patients are usually subjected to high doses over a long time which can eventually increase their side effects.^9,10^ Therefore, there has been an urgent demand for an effective therapeutic approach that can overcome such drawbacks.

Recent developments in the nanotechnology field have facilitated the fabrication of many nanoparticles that can be efficiently exploited for targeted delivery of several drugs. Polymeric nanocarriers have particularly shown good biocompatibility and several other characteristics that include the ability to tailor and functionalize their structures, obtaining tissue-targeted, effective, and safe nanodelivery systems that can be further administered for brain delivery.^11,12^ In this review, a brief introduction to PD and its current pharmacological agents and their limitations will be provided. The review will primarily focus on polymeric-nanotherapeutic drug delivery systems that have recently been utilized for dopamine and levodopa replacement in PD.

## 2. Parkinson's disease

PD has one of the worst growth-rate profiles worldwide, where its fast-paced incidences of prevalence, disability, and mortality are inevitable. In 2016, 6.1 million patients were diagnosed with PD compared to 2.5 million cases in 1990.^13^ Furthermore, more than 200 thousand deaths were reported in 2016, where most of the diagnosed cases have a short average age duration of 15 years.^13,14^

**Fig. 2:**
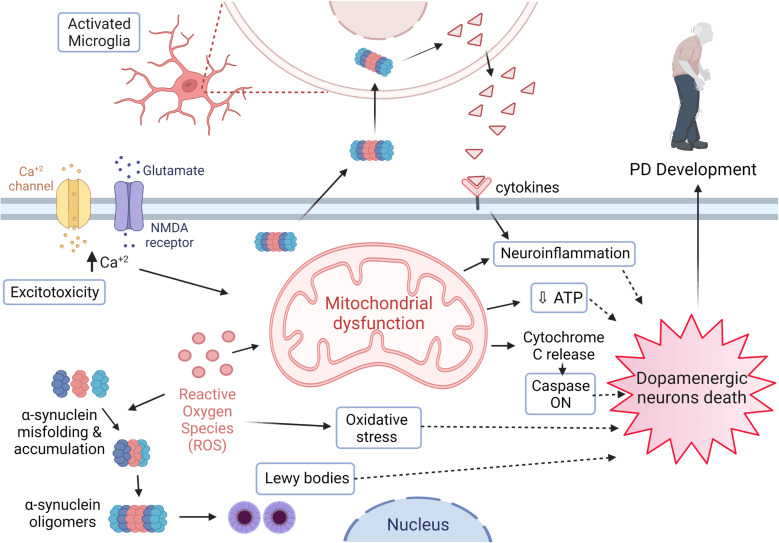
PD pathogenesis. Several neuropathological mechanisms are involved in PD development which eventually result in the death of dopaminergic neurons, and further include microglial activation, α-synuclein misfolding and handling disruption. These cause protein agglomeration and Lewy bodies formation, ROS and oxidative stress, calcium channel malfunction, mitochondrial dysfunction, neuroinflammation, apoptosis, inflammatory markers eruption involving cytokines and other inflammatory markers, and activation of certain Caspase pathways. Figure drawn using Biorender.

PD is a multifactorial disease with risk factors and etiologies that generally involve environmental and genetic ones. Age is considered one of the most significant risk factors related to the PD diagnosis and the onset of its symptoms.^15,16^ Various brain regions are affected in association with the neurodegeneration process as part of the PD pathological progress. However, dopaminergic neurons' loss of the SNpc represents the main cause for the movement disorder among patients. Moreover, Lewy bodies and neurites are the essential pathological hallmarks demonstrated and formed due to the intracellular aggregation of α-synuclein^17–19^ (Fig. 2).

## 3. Current therapeutic approaches to manage PD symptoms

Therapeutic agents have been under development for decades which aim to slow the fast-paced progression of the disease. They are only concerned with its symptoms and deliver only a partial relief. Such therapies have several adverse effects which worsen as the disease develops.^20,21^ Dopaminergic medications represent the majority of drugs prescribed for patients since most PD symptoms develop due to the DA loss. These medications are intended to either imitate the actions of DA or replenish the DA neurotransmitter itself. Hence, they improve the muscles' movement coordination, reducing the rigidity of the involved muscles and the frequency of the tremors.^22^

The gold standard of treatment for PD symptoms is levodopa (l-Dopa), a dopamine precursor drug.^23,24^l-Dopa, unlike DA, can pass the BBB and convert into DA. Since l-Dopa is prone to extensive peripheral metabolism once orally administered before reaching the BBB, it is usually coupled with Carbidopa or benserazide (Dopa decarboxylase inhibitors, DDC-I)^25^ to curb the drug's decarboxylation into DA in the systemic circulation, maximizing l-Dopa's amount delivered to the CNS with fewer side effects in the short-term of treatment. However, the extensive peripheral metabolism of l-Dopa induces several adverse effects such as drowsiness, sleepiness, nausea, and, more importantly, dyskinesia, especially during long-term treatment.^26–28^ Moreover, the chronic oral exposure to l-Dopa coupled with DDC-I was reported to elevate the homocysteine levels due to the increased oxidative stress generation and the diminishing in methylation capacity, resulting in slow and even acute onset of axonal polyneuropathy.^25^ Hence, PD patients with long-term treatment of l-Dopa coupled with DDC-I are eventually vulnerable to extensive motor fluctuations, making such a combination ineffective for continuous dopaminergic stimulation.^25^ Orally inhaled l-Dopa helps overcome the drawbacks associated with oral l-Dopa, especially peripheral l-Dopa decarboxylation. Upper respiratory tract infections, nausea, cough, and discolored sputum are among the common side effects.^29^

DA agonists act on DA receptors, leading to their activation and DA release.^30^ On the other hand, anticholinergics, such as trihexyphenidyl and benztropine, activate DA receptors leading to DA release.^31,32^ Selective catechol-*O*-methyl transferase (COMT) inhibitors can mainly suppress the peripheral degradation of the l-Dopa drug to enhance the BAV of l-Dopa, whereas monoamine oxidase B (MAO-B) inhibitors primarily curb the l-Dopa and DA degradation in the brain.^33–35^ Amantadine augments the dopaminergic responses in the CNS through the initiation of DA and norepinephrine release and the inhibition of their reuptake.^29,36^ Adenosine A2A receptor antagonists (Istradefylline) can curb the over activity of striatopallidal neurons and lower the excessive inhibition of globus pallidus externus gabaminergic (GABAergic) neurons, exerting the antiparkinsonian activity.^29^ Current PD therapeutic drugs and their biological limitations and side effects are summarized in Table 1.

**Table 1:** Biological limitations and side effects of the current PD therapeutic drugs

Drug	Biological limitations	Side/adverse effects reported	Ref.
DA	Hydrophilic in nature	Nausea	37 and 38
	Cannot cross the BBB	Dyskinesia	
	Short plasma half-life	Drowsiness	
	Poor BAV		
l-Dopa	Short half-life	Severe dyskinesia	37 and 39
	Poor pharmacokinetic profile	Nausea	
	Poor BAV (less than 1% can reach the brain milieu)	Drowsiness	
		Motor fluctuations	
DA agonists	Poor pharmacokinetic profile	Repetitive behavior	9 and 40–42
	Poor BAV	Somnolence	
		Cardiac-valvulopathy	
		Legs swelling	
		Retroperitoneal fibrosis	
Anticholinergics	No direct action on the dopaminergic system, where they exert their action only on acetylcholine (muscarinic) receptors	Constipation, confusion, blurred vision, urinary retention, hallucination, drowsiness, tachycardia, dementia, cognitive deficits, *etc.*	31, 32, 43 and 44
		Sudden withdrawal causes the PD symptoms to appear rapidly	
MAO-B/COMT inhibitors	Poor pharmacokinetic profile	Constipation	9, 33–35 and 40
	Poor BAV	Nausea	
	Unable to cross the BBB	Mild hallucinations	
		Dry mouth, *etc.*	
Amantadine	Primarily renally cleared	Insomnia, nausea, hallucination, dizziness, headache, confusion, orthostatic hypotension, peripheral edema, and impulse control disorders	29, 36 and 45
	Close-monitor with renal impairment and avoided in severe cases		
Adenosine A2A receptor antagonist (Istradefylline)	Exclusively eliminated *via* hepatic metabolism	Insomnia, nausea, hallucination, dizziness, constipation, and dyskinesia	29
	Close-monitor with hepatic impairment and avoided in severe cases		

## 4. Nanosystems for drug delivery into the CNS

The chemical and physical properties of drugs can be modified to enhance their BAV and biocompatibility *via* different techniques offered by the nanotechnology field. Such modifications could be performed by incorporating the drug of interest into certain delivery systems designed to reach its site of action. The BBB in the CNS could prevent several medications from achieving their optimal therapeutic efficacy.^46,47^ In such circumstances, the BBB has some strict conditions to protect the brain and allow specific drug molecules to pass to the brain parenchyma; these conditions include a concentration-dependent, selective, and unidirectional permeability for such molecules.^48^ Furthermore, while hydrophobic and small drug molecules effortlessly cross the BBB *via* passive diffusion, large drug molecules and hydrophilic ones cannot cross the BBB without being transported on specific carriers. Hence, such a barrier limitation is believed to be responsible for the failure of DA delivery to the CNS.^49,50^ Conventional therapies for neurodegenerative diseases, on the other hand, fail to reach their site of action or only do so at their lowest concentrations, and they may have further lower or minimal efficacy at greater doses or after an extended period of administration. Moreover, some drug molecules might attach to their peripheral targets leading to adverse effects.^51^

Auspiciously, several nanoparticulate systems have enabled the transportation of drug molecules to reach their specific targets in the brain, revealing an outstanding improvement in their BAV and efficacy with a sustainable manner of drug release at their site of action.^52,53^ Depending on the fluctuations in their gradient concentration, such nanoparticles are believed to be absorbed into the brain and enter the brain parenchyma *via* endocytosis; consequently, the active ingredient is released into the neuron cells or transported across the BBB, reaching the neurons *via* transcytosis.^54^ In addition, the ability to functionalize the surface of various nanocarriers, using particular ligands and peptides, can facilitate the transportation and deliver the drug molecules to their target site through the BBB *via* receptor-mediated active carriers; hence, the drug amounts needed to achieve the optimum therapeutic efficiency can be reduced as well as the cellular uptake will be maximized compared to the conventional free dosage forms of the same drugs.^52,53,55^

Several organic (polymers, lipids, *etc.*), inorganic (metals, zeolites, carbon nanotubes, *etc.*), and hybrid (metal organic frameworks) matrices have been utilized to construct different nanocarriers which then can be used to deliver drugs to specific biological targets. The biodegradable characteristics of organic nanocarriers and polymeric and lipid matrices, make them more preferred to design nanodrug delivery systems.^56,57^ Because of their biocompatibility, polymers have been reported to be used as implants, substrates, and insulating materials for neurological interfaces, where they do not trigger detrimental biological interruptions. Furthermore, different clinical applications have been documented to introduce and benefit from several devices that are formed of polymers, such as biofilms, microspheres, gels, *etc.*^58,59^

## 5. DA and l-Dopa replacement in PD using polymeric nanodelivery systems

Polymeric nanocarriers are stable, biodegradable, offer a sustainable drug release profile, can easily be modified to facilitate their attachments to their corresponding ligands, and can carry high doses of drug molecules. Additionally, the safe profile of several polymers such as polyglycolic acid (PGA), polylactic acid (PLA), and poly lactic-*co*-glycolic acid (PLGA) has supported their widespread usage in many medical applications.^60^ Several studies have been carried out to deliver exogenous DA and l-Dopa into the CNS in order to replace the diminished amounts of the endogenous DA and compensate for the loss of dopaminergic neurons, where advanced polymeric nanoparticulate formulations could have the capability to enhance the PD symptoms *via* delivery of DA into the brain (Table 2).

**Table 2:** The polymeric nanoparticulate systems utilized to replace DA/l-Dopa in PD

Polymeric nanoparticulate system	Therapeutic agent loaded	Route of administration	Particle size (nm)	Zeta potential (mV)	PDI	Model/cell-line	Remarks	Ref.
PLGA/WGA	l-Dopa	Intranasal	652.0 ± 67.7	−3.15 ± 0.435	0.463 ± 0.115	MPTP-injected mice	Compared to conventional oral/intranasal l-Dopa:	61
							Lower cytotoxicity, higher therapeutic efficacy, and less side effects	
PLGA	DA	Intravenous	120	−2.66	0.104	6-OHDA-injected rats	Compared to free DA:	62 and 63
							Higher plasma concentration and extended half-life	
							Significant reduction in ROS autoxidation, dopaminergic neuronal death, and hypersensitivity of D2 receptors	
							Less side effects	
PLGA/albumin	DA	Intraperitoneal	353 to 497	−27 to −37	0.4 to 0.6	6-OHDA-injected mice	Compared to free l-Dopa:	64
							Substantial improvements in motor symptoms	
PEG-*b*-poly(l-Dopa(OAc)_2_)	l-Dopa	Intraperitoneal	∼52.2	—	0.304	MPTP-injected mice	Compared to free l-Dopa:	65
							Sustained release	
							Remarkable enhancement in the *in vivo* pharmacokinetic profile	
							Higher AUC and plasma levels	
							Significant improvement in motor symptoms and dyskinesia suppression	
							Lower cytotoxicity	
Self-assembled neuromelanin inspired polymer/BIX/Fe(AcO)_2_	DA	Intracerebroventricular (i.c.v.) injection/intranasal	(56 to 64) ± 9.0	−13.1	0.124	Healthy adult male Sprague-Dawley rats/6-OHDA-injected rats	Compared to free DA:	66
							Remarkable DA uptake by (BE2-M17) dopaminergic cells	
							Better pharmacokinetic profile	
							Lower cytotoxicity	
							Rapid distribution and higher striatal levels	

Arisoy *et al.* (2020) developed a nanoparticulate delivery-system (*ca.* 652.0 ± 67.7 nm) composed of PLGA (*M*_w_ 7.000–17.000) conjugated with wheat germ agglutinin (WGA) and loaded with l-Dopa which was administered *via* the intranasal route (all mice received 16 mg l-Dopa per kg per day).^61^ Intranasal administration bypasses the BBB; hence, it can be considered an effective route to deliver the polymeric nanosystem directly to the brain. WGA was added to enhance the system adsorption and absorption *via* the nasal cavity and to reduce its elimination.^61^ Moreover, the proposed nanosystem was prepared by the solvent evaporation method of a double emulsion, and its therapeutic efficiency in enhancing the motor deficits was evaluated utilizing an *in vivo* study on an MPTP-induced PD mouse model.^61^ Intranasal administration of the WGA–PLGA nanoparticulate system had shown a significant improvement in the drug delivered to the brain with lower amounts detected in the serum, compared to the conventional drug formulations of l-Dopa administered orally or intranasally, revealing a better therapeutic efficacy and fewer side effects of the nanosystem. Mice treated with the conventional formulation of l-Dopa administered orally showed 0.104 ± 0.007 and 1.868 ± 0.804 ng mL^−1^l-Dopa in serum and brain samples, respectively. Mice treated with l-Dopa administered by the intranasal route had 0.098 ± 0.004 and 2.131 ± 0.254 ng mL^−1^l-Dopa in their serum and brain samples, respectively.^61^ Levels of l-Dopa encapsulated in the WGA–PLGA nanosystem and administered *via* the intranasal route were 0.087 ± 0.004 and 4.177 ± 1.427 ng mL^−1^ in serum and brain samples, respectively.^61^ Moreover, the study revealed better adsorption and tolerability of the nanoparticles throughout the nasal cavity, and lower cytotoxicity had been shown as well (MTT assay conducted on the PC-12 cell line showed no significant cell death after 24 h of exposure to different concentrations, within 6.25–100 μg mL^−1^ of the dose range). The nanosystem had a drug entrapment efficiency of 73% and an extended l-Dopa release of up to 9 h.^61^ These results support the potential of such a delivery system as a promising alternative to free l-Dopa. However, the intranasal route of administration could face some clinical challenges in patients with chronic sinusitis, rhinitis, and other nasal tract infections which could alter the physiological function of the nasal cavity.

Pahuja *et al.* (2015) prepared a polymeric nanoparticulate system composed of PLGA loaded with DA (DA-nanoparticle diameter: *ca.* 120 nm; zeta potential: *ca.* −2.66 mV; polydispersity index (PDI): 0.104; encapsulation efficiency (EE): *ca.* 35.55%; loading efficiency (LE): *ca.* 11.85%).^62,63^ The nanosystem was synthesized using the double emulsion solvothermal method and was reported to enhance the neurological symptoms and animal behavior in 6-hydroxydopamine (6-OHDA)-induced PD model rats with no sudden brain/peripheral changes or heart/blood-pressure abnormalities.^62,63^ In fact, the nanosystem was administered intravenously (4.95 mg DA per kg body weight), and it was further reported to bypass the BBB, reaching the substantia nigra and the striatum and liberating DA in a constant and slow manner.^62,63^ A high concentration of DA (∼45 ng mL^−1^ within 6 h) was delivered compared to <0.5 ng mL^−1^ of the basal DA levels in the rats' plasma.^62,63^ In addition, the nanoparticulate system had shown a significant reduction in the reactive oxygen species (ROS) autoxidation, dopaminergic neuronal death, hypersensitivity of the DA (D2) receptor, and DA plasma clearance from 108.17 to 21.19 mL min^−1^ kg^−1^ after 6 h of the intravenous administration.^62,63^ Furthermore, the DA half-life in the plasma increased from 1.22 h to 2.53 h.^62,63^ Overall, the proposed nanosystem was able to deliver DA to the brain and overcome the side effects associated with the administration of free DA. Further safety investigations might be considered to examine the possibility of accumulation of the nanoparticles in different organ tissues. Additionally, the reported EE and LE of the nanosystem are low and therefore future studies should explore increasing both parameters. Furthermore, clearance of the proposed nanoparticles (which depends on their size, charge, and surface modification and influences their circulation half-life, hepatic filtration and phagocytic uptake) should be investigated.

Monge-Fuentes *et al.* (2021) designed another polymeric nanosystem loaded with DA (7 μg DA per mg of nanoparticles) composed of albumin and PLGA (353 to 497 nm in diameter; PDI: 0.4 to 0.6; zeta potential −27 to −37 mV).^64^ It is of note that albumin is non-immunogenic, biocompatible, can cross the BBB through a specific receptor, and enhances both the stability and half-life of the nanosystem. A 6-OHDA-induced PD mouse model had been utilized to evaluate the nanosystem’s neurotherapeutic efficacy. Substantial improvements in motor symptoms were reported compared to those in lesioned animals and those receiving free l-Dopa.^64^ This nanosystem was able to cross the BBB and recompensate the diminished levels of DA in the substantia nigra and also promoted (at a dose of 20 mg per animal) the sensorimotor, motor coordination, and balance performance to levels seen in non-lesioned animals.^64^

**Fig. 3:**
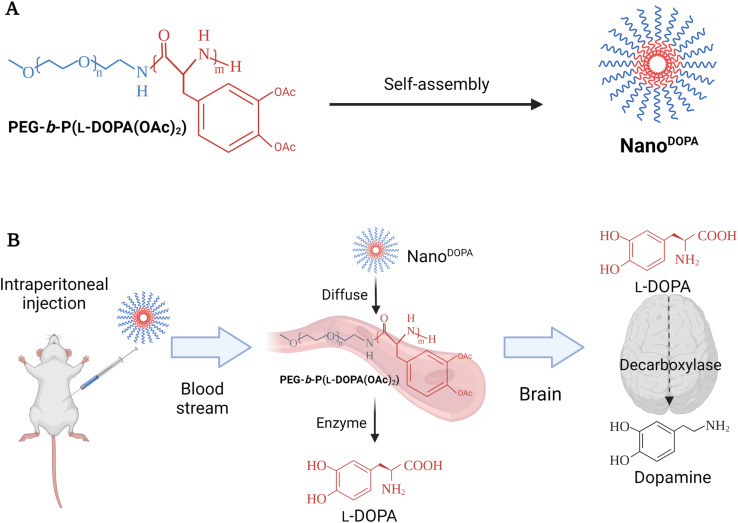
The figure briefly reveals the formation and the delivery strategy of Nano^DOPA^, a nano-polymeric-based-drug system, which was primarily designed to enhance the brain-delivery and the therapeutic efficiency of l-Dopa in a PD mouse model. Nano^DOPA^ was formed after the self-assembly of the copolymer, PEG-*b*-poly(l-Dopa(OAc)_2_), in the form of micelles in an aqueous environment, and the size range of these self-assembled micelles reached a few tens of nanometers. (A) The structure and preparation design of Nano^DOPA^. (B) The drug-delivery strategy utilized to replace DA in PD mouse model brains through intraperitoneal drug injection. Reproduced with permission from ref. 65. Figure drawn using Biorender.

BinhVong *et al.* (2020) designed a nano-polymeric-based-drug system (Nano^DOPA^) to enhance the brain-delivery and therapeutic efficiency of l-Dopa in a PD mouse model.^65^ Briefly, Nano^DOPA^ was formed after the self-assembly of the copolymer, PEG-*b*-poly(l-Dopa(OAc)_2_), in the form of micelles in an aqueous environment, and the size range of these self-assembled micelles reached a few tens of nanometers (Fig. 3).^65^ The nanodrug was reported to have a concentration of 0.4 mmol l-Dopa per mL with a ∼52.2 nm particle size and PDI of 0.304. Furthermore, the peptide bonds of the lipophilic precursor can be hydrolyzed inside the block-copolymer to give a gradual release of l-Dopa into circulation upon hydrolysis by esterases and proteases.^65^ Furthermore, the Nano^DOPA^ drug was successfully reported to liberate l-Dopa gradually by chymotrypsin degradation, and to significantly enhance the *in vivo* pharmacokinetic profile of l-Dopa when compared to that of free l-Dopa. Both area-under-the-curve (AUC) and plasma level of l-Dopa were increased, following the intraperitoneal injection of Nano^DOPA^ (127 μmol l-Dopa per kg). Plasma levels for free l-Dopa reached 300 ng mL^−1^ after 0.5 h of the injection and returned to the baseline at 1 h, whereas the plasma levels for l-Dopa administered as Nano^DOPA^ were ∼135 ng mL^−1^ at 0.5 h and were maintained up to 12 h. The AUC of plasma from mice treated with free l-Dopa and Nano^DOPA^ was 25.2 ± 2.4 ng h mL^−1^ and 200.7 ± 28.6 ng h mL^−1^, respectively.^65^ Overall, the administration of Nano^DOPA^ in the MPTP-induced PD mouse model led to a remarkable improvement in motor symptoms and suppression of dyskinesia which is usually associated with l-Dopa treatment. The suppression of dyskinesia in treated mice was examined using certain behavioral tests. The mice were administered high doses of l-Dopa (25 mg kg^−1^, equivalent to 127 μmol kg^−1^) or Nano^DOPA^ (105 mg kg^−1^, equivalent to 127 μmol kg^−1^ of l-Dopa) combined with 10 mg kg^−1^ of benserazide. The administration of l-Dopa or Nano^DOPA^ was initiated daily in mice for 18 days starting from day 1, after the intraperitoneal injection of MPTP with a total dose of 170 mg kg^−1^ given at different intervals to induce PD-like symptoms. Consequently, the dyskinesia severity could be assessed by two behavioral tests: the slider test and the abnormal involuntary movement evaluation. For the slider test, the fall latency of mice was recorded after being placed on a slider for 3 min. For the abnormal involuntary movements' evaluation, clear plastic cages were utilized to place each mouse on a cage, where the involuntary movements of each could be recorded, setting a scale from 0 to 4 to rate each mouse on four subscales. The abnormal involuntary movement scale was as follows: 0 = absent, 1 = <50%, 2 = >50%, 3 = continuous unless interrupted by a stimulus, and 4 = continuous. The four subscales were: oral dyskinesia, limb dyskinesia, contraversive rotation, and axial dystonia. The mouse group treated with Nano^DOPA^ had shown a significantly lower score on the abnormal involuntary movement scale than those treated with l-Dopa alone at equivalent doses of l-Dopa (127 μmol kg^−1^). In addition, a remarkable enhancement in the latency time was recorded from the slider test with the Nano^DOPA^ group compared to the l-Dopa and MPTP mouse groups.^65^ Furthermore, no toxicity had been reported in the major organs of the treated mice following long-term administration of high doses of the drug. Using an MTT assay, the IC_50_ values were 0.583 mM and 2.88 mM for free l-Dopa and Nano^DOPA^, respectively.^65^ Such promising results might encourage the development of self-assembled Nano^DOPA^ for the treatment of Parkinson's and other neurodegenerative diseases. However, the intraperitoneal administration of drugs in humans is limited (mainly used for delivering chemotherapeutics against pancreatic and ovarian cancers) and suffers from variable effectiveness and misinjection. Therefore, alternative administration routes should be explored.

**Fig. 4:**
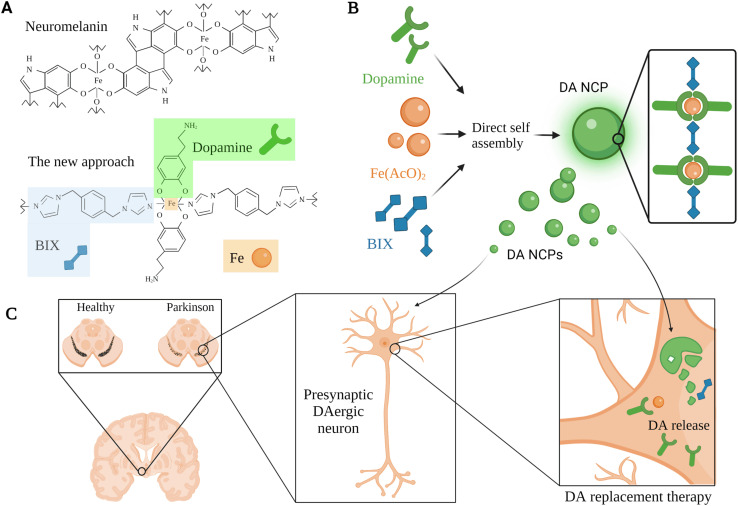
A schematic representation for a DA replacement approach inspired by neuromelanin in PD, showing the multiple steps used for the development of a tailor-made co-ordination polymer and the anticipated biological function. (A) Neuromelanin structural composition and the associated nanoscale co-ordination polymeric system (DA-NCP). The co-ordination polymer was formed with iron (Fe) nodes in the central backbone of the polymer which were further linked to a bi-dentate ligand (*e.g.*, BIX), and to proceed with the co-ordination sphere formation, a counter ligand such as DA was utilized. (B) The main bio-elements introduced in the polymeric nanoparticulate system were BIX (1,4-bis(imidazol-1-ylmethyl)benzene), Fe(AcO)_2_, and DA. These bio-elements were directly self-assembled to synthesize the DA-NCP nanoparticles. (C) The paradigm for DA replacement in PD using the proposed polymeric nanoparticulate system, where the anticipated biological function of the DA-NCP nanoparticulate system might be shown.^66^ Figure drawn using Biorender.

Inspired by neuromelanin particles, which can be found in dopaminergic neurons and comprise considerable amounts of DA and iron ions, García-Pardo *et al.* (2021) developed a polymeric nanoparticulate system to enrich DA delivery.^66^ This approach aimed to encapsulate DA within polymeric nanoparticles, benefiting from the ability of DA to form a reversible coordination complex with iron metal; hence, DA can be polymerized with a bis-imidazole ligand (BIX) (Fig. 4).^66^ The polymeric nanoparticulate system (56–64 nm; PDI 0.124; EE 52.5 ± 7.2% DA + BIX 20%) was reported to enhance the loading efficiency of DA (69.7 ± 7.2%) and the DA uptake by the dopaminergic cells (BE2-M17).^66^ Administration of 10 μg mL^−1^ polymeric nanoparticles for 2 h induced up to 6360 ng mg^−1^ of DA at the intracellular level compared to 1156 ng mg^−1^ obtained for free DA under the same concentration and conditions.^66^ In addition, the DA release kinetics were improved (50% after 2 h and 5% over the following 22 h, at pH 7.4). The nanoparticles showed lower toxicity compared to free DA with no obvious reduction in BE2-M17 cell viability when treated with up to 100 μg mL^−1^ of the nanoparticles, whereas a slightly higher concentration of free DA led to the death of >85% of dopaminergic cells.^66^ Moreover, rapid distribution and higher striatal DA levels (>2500 ng in ipsilateral and >5000 ng in the contralateral side after 2 h) were shown when the nanoparticulate system was directly infused *in vivo* in healthy rats' ventricles.^66^ In contrast, the administration *via* the nasal route of a dose equivalent to 200 μg of free DA daily after four days revealed a significant reduction in the duration and the number of apomorphine-induced rotations compared to those observed in controls (both vehicle and DA treated rats).^66^ This polymeric nanostructure might be further exploited for DA replacement with some concerns that could be subsided in the future related to the nasal route limitations mentioned above for the clinical application.

The outstanding properties of chitosan nanoparticles have enabled their utilization for different drug delivery systems, where they have shown several advantages such as ease of preparation and functionalization, biocompatibility, *etc.* Ragusa *et al.* (2018) had successfully prepared chitosan nanoparticles which encapsulate DA and tested the neuroprotective effects of the system, *in vitro*, on human neuroblastoma SH-SY5Y cells.^67^ They further studied the various effects of DA, bare chitosan nanoparticles, and chitosan-dopamine nanoparticles in producing ROS.^67^ The chitosan-DA nanoparticles had an average size of 109 ± 16 nm, a surface charge of +34 ± 0.9 mV, a PDI of 0.4, a DA concentration of 5 mg mL^−1^, and a DA LE% of 64%. DA encapsulation, in the proposed nanoparticulate system, and its subsequent sustained release (almost 50% of DA released after 48 h) had greatly lowered the *in vitro* oxidation rate (lowered the H_2_O_2_ induction rate compared to that induced by the same concentration of free DA), suggesting that it may have neuroprotective properties.^67^ H_2_O_2_ generated by SH-SY5Y cell lines after incubation with 100 μM of free DA or the nanosystem reached ∼10 and ∼6 mg H_2_O_2_ per mg protein, respectively.^67^ However, the MTT assay test using the SH-SY5Y cell line, showed 90% and 75% cell viability upon treatment with a 100 μM concentration of free DA or the nanosystem, respectively.^67^ Further examination of the enzymatic activity induced by the chitosan-dopamine nanoparticulate system revealed that both superoxide dismutase (SOD) and glutathione peroxidase (GPx) enzymes might greatly contribute to the oxidative stress mitigation.^67^ At the same 100 μM concentration of free DA and chitosan-dopamine nanoparticles, the nanosystem showed ∼11 units per mg protein of SOD activity compared to ∼8 units per mg protein for the free DA, whereas the nanosystem showed ∼0.11 units per mg protein of GPx activity compared to ∼0.075 units per mg protein for the free DA.^67^ Overall, compared to free DA, the chitosan-DA nanoparticles had shown lower cytotoxicity and lower oxidative stress against human neuroblastoma SH-SY5Y cells in addition to higher SOD and GPx enzymatic activities and sustainable release of DA.^67^ Although chitosan nanoparticles encapsulating DA had shown promising *in vitro* neuroprotective effects, *in vivo* studies are needed to support future clinical application.

## 6. Conclusions and future trends

PD is a multi-factorial disorder requiring a variety of combination therapies to alleviate its symptoms and curb its fast-paced progression. Although l-Dopa has been considered the gold-standard therapy, its poor BAV and higher administration frequency can ultimately worsen some already existing symptoms. The transportation across the BBB also represents an additional challenge in treatment of PD patients. Therefore, it is necessary to develop biocompatible delivery systems that can overcome the drawbacks mentioned earlier to improve the delivery of DA/l-Dopa in the CNS, providing better outcomes to PD patients.

Polymeric-based nanodrug delivery systems have shown advantages such as biocompatibility and biodegradability. Additionally, they can be tailored and functionalized to specifically reach their target tissues. PLGA/WGA and PEG-*b*-poly(l-Dopa(OAc)_2_) are two polymeric nanoparticulate systems used to deliver l-Dopa utilizing PLGA and PEG polymers. Both approaches showed significant improvements in the pharmacokinetic profile of l-Dopa, lower cytotoxicity, better therapeutic efficiency, enhanced delivery across the BBB, minor side effects, greater motor symptom enhancement and dyskinesia suppression compared to free l-Dopa.^61,65^ Other polymeric nanocarriers that had been successfully used to deliver DA are PLGA, PLGA/albumin, self-assembled neuromelanin/BIX/Fe(AcO)_2_, and chitosan polymers. These delivery systems resulted in higher DA concentration delivery with better conservation of the dopaminergic neuronal cells and reduced ROS autoxidation and other side effects associated with free DA. Also, both the release and stability profiles of DA were greatly enhanced with substantial improvements in symptoms.^62–64,66,67^ Overall, such polymeric nanocarriers have been suggested as promising drug delivery systems with their outstanding therapeutic benefits in improving motor symptoms and other manifestations of PD, reflecting an encouraging possibility for their future utilization as drug nanocarriers for treatment of neurodegenerative diseases.

While it is well established that the BBB characteristics are significantly altered in *in vivo* Parkinsonian models and other neurodegenerative disease models,^68^ the chemical–physical properties of the nanoparticles that have been utilized so far have not yet been assessed for their ability to cross the BBB and target dopaminergic cells in humans. So far, no clinical trials of a nanoparticulate system have been conducted on PD patients.

For clinical applications, safety concerns should also be investigated. For example, the intravenous delivery of nanoparticulate systems would lead to their accumulation in the kidneys, liver, and spleen. As a result, it is crucial to establish nano-formulations that might be remotely prompted to release their loaded medication upon reaching their target inside the brain. Moreover, although polymeric nanoparticles have established biocompatibility, toxicity still should be assessed as it depends on several parameters such as the size, charge, type, and composition of the polymer used.

The BBB has been reported to be altered in PD patients.^68^ The hallmark of this alteration is the increased leakage which impairs the ability of the BBB to prevent harmful molecules from entering the brain. Therefore, novel nano-delivery systems especially those capable of exploiting these alterations should be developed for the targeted delivery of promising therapeutics and imaging agents. Such systems could have great potential as efficient therapeutics for PD and neurodegenerative diseases.

## Conflicts of interest

The authors declare that they have no known competing financial interests or personal relationships that could have appeared to influence the work reported in this paper.

## Supplementary Material

## References

[cit1] Duty S., Jenner P. (2011). Animal models of Parkinson's disease: a source of novel treatments and clues to the cause of the disease. Br. J. Pharmacol..

[cit2] Jagmag S. A., Tripathi N., Shukla S. D., Maiti S., Khurana S. (2016). Evaluation of models of Parkinson's disease. Front. Neurosci..

[cit3] De Virgilio A., Greco A., Fabbrini G., Inghilleri M., Rizzo M. I., Gallo A., Conte M., Rosato C., Appiani M. C., De Vincentiis M. (2016). Parkinson's disease: autoimmunity and neuroinflammation. Autoimmun. Rev..

[cit4] Mohamad K. A., El-Naga R. N., Wahdan S. A. (2022). Neuroprotective effects of indole-3-carbinol on the rotenone rat model of Parkinson's disease: Impact of the SIRT1-AMPK signaling pathway. Toxicol. Appl. Pharmacol..

[cit5] Lang A. E., Obeso J. A. (2004). Challenges in Parkinson's disease: restoration of the nigrostriatal dopamine system is not enough. Lancet Neurol..

[cit6] Chaudhuri K. R., Healy D. G., Schapira A. H. (2006). Non-motor symptoms of Parkinson's disease: diagnosis and management. Lancet Neurol..

[cit7] Ohl F., Arndt S. S., van der Staay F. J. (2008). Pathological anxiety in animals. Vet. J..

[cit8] Chaudhuri K. R., Schapira A. H. (2009). Non-motor symptoms of Parkinson's disease: dopaminergic pathophysiology and treatment. Lancet Neurol..

[cit9] Deleu D., Northway M. G., Hanssens Y. (2002). Clinical pharmacokinetic and pharmacodynamic properties of drugs used in the treatment of Parkinson's disease. Clin. Pharmacokinet..

[cit10] Pires A. O., Teixeira F. G., Mendes-Pinheiro B., Serra S. C., Sousa N., Salgado A. J. (2017). Old and new challenges in Parkinson's disease therapeutics. Prog. Neurobiol..

[cit11] Begines B., Ortiz T., Pérez-Aranda M., Martínez G., Merinero M., Argüelles-Arias F., Alcudia A. (2020). Polymeric nanoparticles for drug delivery: Recent developments and future prospects. Nanomaterials.

[cit12] Saraiva C., Praça C., Ferreira R., Santos T., Ferreira L., Bernardino L. (2016). Nanoparticle-mediated brain drug delivery: overcoming blood–brain barrier to treat neurodegenerative diseases. J. Controlled Release.

[cit13] Dorsey E. R., Elbaz A., Nichols E., Abd-Allah F., Abdelalim A., Adsuar J. C., Ansha M. G., Brayne C., Choi J.-Y. J., Collado-Mateo D. (2018). Global, regional, and national burden of Parkinson's disease, 1990–2016: a systematic analysis for the Global Burden of Disease Study 2016. Lancet Neurol..

[cit14] Elbaz A., Carcaillon L., Kab S., Moisan F. (2016). Epidemiology of Parkinson's disease. Rev. Neurol..

[cit15] Przedborski S. (2017). The two-century journey of Parkinson disease research. Nat. Rev. Neurosci..

[cit16] Bose A., Beal M. F. (2016). Mitochondrial dysfunction in Parkinson's disease. J. Neurochem..

[cit17] Schrag A., Horsfall L., Walters K., Noyce A., Petersen I. (2015). Prediagnostic presentations of Parkinson's disease in primary care: a case-control study. Lancet Neurol..

[cit18] Dipiro J. T. , Talbert R. L., Yee G. C., Matzke G. R., Wells B. G. and Posey L. M., Pharmacotherapy: a Pathophysiologic Approach, McGraw-Hill Medical, New York, 2014

[cit19] Kouli A. , Torsney K. M. and Kuan W.-L., Parkinson's Disease: Etiology, Neuropathology, and Pathogenesis, Exon Publications, 2018, pp. 3–26, 10.15586/codonpublications.parkinsonsdisease.2018.ch1 30702842

[cit20] Stoker T. B. and Greenland J. C., Parkinson's Disease: Pathogenesis and Clinical Aspects [internet], 2018, 10.15586/codonpublications.parkinsonsdisease.2018 30702835

[cit21] Oertel W., Schulz J. B. (2016). Current and experimental treatments of Parkinson disease: a guide for neuroscientists. J. Neurochem..

[cit22] Singh N., Pillay V., Choonara Y. E. (2007). Advances in the treatment of Parkinson's disease. Prog. Neurobiol..

[cit23] Ellis J. M., Fell M. J. (2017). Current approaches to the treatment of Parkinson's Disease. Bioorg. Med. Chem. Lett..

[cit24] Jankovic J., Aguilar L. G. (2008). Current approaches to the treatment of Parkinson's disease. Neuropsychiatr. Dis. Treat..

[cit25] Müller T. (2020). Pharmacokinetics and pharmacodynamics of levodopa/carbidopa cotherapies for Parkinson's disease. Expert Opin. Drug Metab. Toxicol..

[cit26] Bozdağ Pehlivan S. (2013). Nanotechnology-based drug delivery systems for targeting, imaging and diagnosis of neurodegenerative diseases. Pharm. Res..

[cit27] Yang X., Zheng R., Cai Y., Liao M., Yuan W., Liu Z. (2012). Controlled-release levodopa methyl ester/benserazide-loaded nanoparticles ameliorate levodopa-induced dyskinesia in rats. Int. J. Nanomed..

[cit28] Stocchi F., Rascol O., Kieburtz K., Poewe W., Jankovic J., Tolosa E., Barone P., Lang A. E., Olanow C. W. (2010). Initiating levodopa/carbidopa therapy with and without entacapone in early Parkinson disease: the STRIDE-PD study. Ann. Neurol..

[cit29] Aradi S. D., Hauser R. A. (2020). Medical management and prevention of motor complications in Parkinson's disease. Neurotherapeutics.

[cit30] Chondrogiorgi M., Tatsioni A., Reichmann H., Konitsiotis S. (2014). Dopamine agonist monotherapy in Parkinson's disease and potential risk factors for dyskinesia: a meta-analysis of levodopa-controlled trials. Eur. J. Paediatr. Neurol..

[cit31] Sheu J. J., Tsai M. T., Erickson S. R., Wu C. H. (2019). Association between anticholinergic medication use and risk of dementia among patients with Parkinson's disease. Pharmacotherapy.

[cit32] Crispo J. A., Willis A. W., Thibault D. P., Fortin Y., Hays H. D., McNair D. S., Bjerre L. M., Kohen D. E., Perez-Lloret S., Mattison D. R. (2016). Associations between anticholinergic burden and adverse health outcomes in Parkinson disease. PLoS One.

[cit33] Fahn S. (2015). The medical treatment of Parkinson disease from James Parkinson to George Cotzias. Mov. Disord..

[cit34] Finberg J. P. (2019). Inhibitors of MAO-B and COMT: Their effects on brain dopamine levels and uses in Parkinson's disease. J. Neural Transm..

[cit35] Connolly B. S., Lang A. E. (2014). Pharmacological treatment of Parkinson disease: a review. JAMA, J. Am. Med. Assoc..

[cit36] Elmer L. W., Juncos J. L., Singer C., Truong D. D., Criswell S. R., Parashos S., Felt L., Johnson R., Patni R. (2018). Pooled analyses of phase III studies of ADS-5102 (amantadine) extended-release capsules for dyskinesia in Parkinson's disease. CNS Drugs.

[cit37] You H., Mariani L.-L., Mangone G., de Nailly D. L. F., Charbonnier-Beaupel F., Corvol J.-C. (2018). Molecular basis of dopamine replacement therapy and its side effects in Parkinson's disease. Cell Tissue Res..

[cit38] Rautio J., Kumpulainen H., Heimbach T., Oliyai R., Oh D., Järvinen T., Savolainen J. (2008). Prodrugs: design and clinical applications. Nat. Rev. Drug Discovery.

[cit39] Borgkvist A., Avegno E. M., Wong M. Y., Kheirbek M. A., Sonders M. S., Hen R., Sulzer D. (2015). Loss of striatonigral GABAergic presynaptic inhibition enables motor sensitization in parkinsonian mice. Neuron.

[cit40] De Miranda B. R., Miller J. A., Hansen R. J., Lunghofer P. J., Safe S., Gustafson D. L., Colagiovanni D., Tjalkens R. B. (2013). Neuroprotective efficacy and pharmacokinetic behavior of novel anti-inflammatory para-phenyl substituted diindolylmethanes in a mouse model of Parkinson's disease. J. Pharmacol. Exp. Ther..

[cit41] Jain S., Waters C. H. (2007). Controversies of dopamine agonists: somnolence, cardiac valvulopathy and repetitive behaviors. Curr. Drug Ther..

[cit42] Alberti C. (2015). Drug-induced retroperitoneal fibrosis: short aetiopathogenetic note, from the past times of ergot-derivatives large use to currently applied bio-pharmacology. Il Giorn. Chir..

[cit43] Hong C.-T., Chan L., Wu D., Chen W.-T., Chien L.-N. (2019). Antiparkinsonism anticholinergics increase dementia risk in patients with Parkinson's disease. Parkinsonism Relat. Disord..

[cit44] Rajan R., Saini A., Verma B., Choudhary N., Gupta A., Vishnu V. Y., Bhatia R., Singh M. B., Srivastava A. K., Srivastava M. V. P. (2020). Anticholinergics may carry significant cognitive and gait burden in Parkinson's Disease. Mov. Disord. Clin. Pract..

[cit45] deVries T., Dentiste A., Di Lea C., Pichette V., Jacobs D. (2019). Effects of renal impairment on the pharmacokinetics of once-daily amantadine extended-release tablets. CNS Drugs.

[cit46] Kumar A., Tan A., Wong J., Spagnoli J. C., Lam J., Blevins B. D., Thorne L., Ashkan K., Xie J., Liu H. (2017). Nanotechnology for neuroscience: promising approaches for diagnostics, therapeutics and brain activity mapping. Adv. Funct. Mater..

[cit47] Chowdhury A., Kunjiappan S., Panneerselvam T., Somasundaram B., Bhattacharjee C. (2017). Nanotechnology and nanocarrier-based approaches on treatment of degenerative diseases. Int. Nano Lett..

[cit48] Pardridge W. M. (2005). The blood–brain barrier: bottleneck in brain drug development. NeuroRx.

[cit49] Upadhyay R. K. (2014). Transendothelial transport and its role in therapeutics. Int. Scholarly Res. Not..

[cit50] Rautio J., Laine K., Gynther M., Savolainen J. (2008). Prodrug approaches for CNS delivery. AAPS J..

[cit51] Alavijeh M. S., Chishty M., Qaiser M. Z., Palmer A. M. (2005). Drug metabolism and pharmacokinetics, the blood–brain barrier, and central nervous system drug discovery. NeuroRx.

[cit52] Yemisci M., Caban S., Gursoy-Ozdemir Y., Lule S., Novoa-Carballal R., Riguera R., Fernandez-Megia E., Andrieux K., Couvreur P., Capan Y. (2015). Systemically administered brain-targeted nanoparticles transport peptides across the blood—brain barrier and provide neuroprotection. J. Cereb. Blood Flow Metab..

[cit53] Zensi A., Begley D., Pontikis C., Legros C., Mihoreanu L., Wagner S., Büchel C., von Briesen H., Kreuter J. r. (2009). Albumin nanoparticles targeted with Apo E enter the CNS by transcytosis and are delivered to neurones. J. Controlled Release.

[cit54] Lockman P., Mumper R., Khan M., Allen D. (2002). Nanoparticle technology for drug delivery across the blood–brain barrier. Drug Dev. Ind. Pharm..

[cit55] Blanco E., Shen H., Ferrari M. (2015). Principles of nanoparticle design for overcoming biological barriers to drug delivery. Nat. Biotechnol..

[cit56] Jesus M. and Grazu V., Nanobiotechnology: Inorganic Nanoparticles *vs* Organic Nanoparticles, Elsevier, 2012

[cit57] Medina C., Santos-Martinez M., Radomski A., Corrigan O., Radomski M. (2007). Nanoparticles: pharmacological and toxicological significance. Br. J. Pharmacol..

[cit58] Ramasamy M., Lee J. (2016). Recent nanotechnology approaches for prevention and treatment of biofilm-associated infections on medical devices. BioMed Res. Int..

[cit59] Hassler C., Boretius T., Stieglitz T. (2011). Polymers for neural implants. J. Polym. Sci., Part B: Polym. Phys..

[cit60] Pethe A. M., Yadav K. S. (2019). Polymers, responsiveness and cancer therapy. Artif. Cells, Nanomed., Biotechnol..

[cit61] Arisoy S., Sayiner O., Comoglu T., Onal D., Atalay O., Pehlivanoglu B. (2020). *In vitro* and *in vivo* evaluation of levodopa-loaded nanoparticles for nose to brain delivery. Pharm. Dev. Technol..

[cit62] Pahuja R., Seth K., Shukla A., Shukla R. K., Bhatnagar P., Chauhan L. K. S., Saxena P. N., Arun J., Chaudhari B. P., Patel D. K. (2015). Trans-blood brain barrier delivery of dopamine-loaded nanoparticles reverses functional deficits in parkinsonian rats. ACS Nano.

[cit63] Pahuja R., Seth K., Shukla A., Shukla R. K., Bhatnagar P., Chauhan L. K. S., Saxena P. N., Arun J., Chaudhari B. P., Patel D. K. (2019). Correction to trans-blood brain barrier delivery of dopamine-loaded nanoparticles reverses functional deficits in Parkinsonian Rats. ACS Nano.

[cit64] Monge-Fuentes V., Mayer A. B., Lima M. R., Geraldes L. R., Zanotto L. N., Moreira K. G., Martins O. P., Piva H. L., Felipe M. S. S., Amaral A. C. (2021). Dopamine-loaded nanoparticle systems circumvent the blood–brain barrier restoring motor function in mouse model for Parkinson's Disease. Sci. Rep..

[cit65] Vong L. B., Sato Y., Chonpathompikunlert P., Tanasawet S., Hutamekalin P., Nagasaki Y. (2020). Self-assembled polydopamine nanoparticles improve treatment in Parkinson's disease model mice and suppress dopamine-induced dyskinesia. Acta Biomater..

[cit66] García-Pardo J., Novio F., Nador F., Cavaliere I., Suárez-García S., Lope-Piedrafita S., Candiota A. P., Romero-Gimenez J., Rodríguez-Galván B., Bové J. (2021). Bioinspired Theranostic Coordination Polymer Nanoparticles for Intranasal Dopamine Replacement in Parkinson's Disease. ACS Nano.

[cit67] Ragusa A., Priore P., Giudetti A. M., Ciccarella G., Gaballo A. (2018). Neuroprotective investigation of chitosan nanoparticles for dopamine delivery. Appl. Sci..

[cit68] Pan Y., Nicolazzo J. A. (2018). Impact of aging, Alzheimer's disease and Parkinson's disease on the blood–brain barrier transport of therapeutics. Adv. Drug Delivery Rev..

[cit69] Bridi J. C., Hirth F. (2018). Mechanisms of α-synuclein induced synaptopathy in Parkinson's disease. Front. Neurosci..

